# Assessing the analgesic efficacy of oral epigallocatechin-3-gallate on epidural catheter analgesia in patients after surgical stabilisation of multiple rib fractures: a prospective double-blind, placebo-controlled clinical trial

**DOI:** 10.1080/13880209.2020.1797123

**Published:** 2020-08-04

**Authors:** Lihong Zhang, Weifeng Liu, Haiping You, Zhiyuan Chen, Liming Xu, Hefan He

**Affiliations:** Department of Anesthesiology, Second Affiliated Hospital, Fujian Medical University, Quanzhou, China

**Keywords:** Pain relief, incentive spirometry, respiratory rate, oxygen saturation

## Abstract

**Context:**

Thoracic trauma results in multiple rib fractures (MRF), and surgical stabilisation of rib fractures (SSRF) can relieve fracture pain. Epigallocatechin-3-gallate (EGCG) is reported to exhibit beneficial effects in bone-related metabolic and differentiation processes.

**Objective:**

To study the clinical effect of EGCG on regional analgesia for pain relief in MRF patients after SSRF.

**Materials and methods:**

Ninety-seven MRF patients (61 males, 36 females) who were on epidural catheter analgesia after SSRF were recruited. They were randomly divided into: oral EGCG 100 mg (oral grade) twice daily for 10 days and placebo groups. Pain scores, incentive spirometry (IS) volumes, respiratory rate and oxygen saturation (SpO_2_) were assessed day 10 after SSRF.

**Results:**

Comparing results from the placebo and EGCG group, in the 10-day intervention course, oral EGCG reduced pain score (8 at base line vs. 4 at end of intervention in EGCG group, *p* < 0.05; 4 in EGCG group vs. 6 in placebo group at end of intervention, *p* < 0.05), improved IS volume (713 at base line vs. 1072 at end of intervention in EGCG group, *p* < 0.05; 1072 in EGCG group vs. 953 in placebo group at end of intervention, *p* < 0.05) and respiratory rate (24 at base line vs. 15 at end of intervention in EGCG group, *p* < 0.05; 15 in EGCG group vs. 19 in placebo group at end of intervention, *p* < 0.05). However, no further enhancing effect on SpO_2_ was observed in the EGCG group (0.98 in EGCG group vs. 0.98 in placebo group at end of intervention, *p* > 0.05).

**Discussion and conclusions:**

Although the study is limited by a relatively small sample size and lack of serum factor analysis, the key results and the study design, for the first time, nevertheless pave the way for trials with larger number of patients to understand the effect of EGCG in MRF patients that are undergoing SSRF.

## Introduction

Thoracic trauma frequently results in multiple rib fractures (MRF), which occur in over 60% of all thoracic trauma incidences (Simon et al. 2005). Based on statistical reports, 300,000 patients suffered from rib fractures in 2004 in the USA alone (Lafferty et al. 2011), and increased to over 350,000 cases in 2017 (Pieracci et al. 2017). Surgical stabilisation of rib fractures (SSRF) can prevent rib shortening and displacement, relieve fracture pain, and reduce the risk of non-union. Importantly, SSRF can improve ventilation, reduce dependence on ventilators and the incidences of complications such as pneumonia, length of hospital stay, and rates of mortality and disability (Solberg et al. 2009; Fraser et al. 2017). MRF are commonly seen in injuries caused by high energy mechanisms (Haenel et al. 1995). Pain, as a result of MRF, is mainly associated with decreased respiratory effort, leading to failure to clear secretions, atelectasis, and reduced vital capacity. Necessary pain management for MRF not only provides relief of symptoms, but also prevents secondary respiratory complications and decreases splinting (Fusco et al. 2017).

In MRF patients, local/regional analgesic techniques, compared to systemic analgesia, have been demonstrated to yield greater efficacy and fewer side effects (Karmakar & Ho 2003; Ho et al. 2011). Epidural (EPI) catheter analgesia is currently the most frequently studied regional technique (Lynch et al. 2019), and is recommended by the Eastern Association for the Surgery of Trauma and Trauma Anaesthesiology Society (Galvagno et al. 2016). However, the studies associated with EPI analgesia have been controversial (Lynch et al. 2019), thus warranting additional investigations to identify novel agents for further pain relief, especially in MRF patients after SSRF.

Green tea is a non-fermented and non-oxidized product containing several polyphenolic catechins, with epigallocatechin-3-gallate (EGCG) as one of the most biologically potent components (Liao et al. 2001). Various reports have suggested the beneficial effects of EGCG in bone-related metabolic and differentiation processes (Shen et al. 2009). For instance, EGCG could promote healing of femoral bone defects (Lin et al. 2019). Oxidative EGCG coating on polymeric substrates has been demonstrated to regulate activities of multiple cell types that are beneficial for healing of bone fractures (Madhurakkat Perikamana et al. 2019). However, the role of EGCG in pain management of MRF has yet to be investigated.

In the current clinical study, we recruited MRF patients, who were on EPI catheter analgesia after SSRF, and administered oral EGCG, followed by assessing their pain scores, incentive spirometry (IS) volumes, respiratory rates, and oxygen saturation (SpO_2_). The objective of the study was to assess the analgesic efficacy of oral EGCG in MRF patients who were on EPI catheter analgesia after SSRF.

## Patients and methods

### Patients

All patients were recruited from those admitted into the Emergency Department of Second Affiliated Hospital, Fujian Medical University for MRF. This clinical trial was approved by the Ethics Review Board of Second Affiliated Hospital, Fujian Medical University, and registered with Chinese Clinical Trial Registry (Clinical Trial Registration Number ChiCTR1900028247). All recruited patients have given written informed consent forms, and their data were completely de-identified to keep their confidentiality.

The study population consisted of 97 MRF patients who underwent complete evaluation including chest 3 D-computed tomography and met the inclusion and exclusion criteria for undergoing SSRF in Second Affiliated Hospital, Fujian Medical University from January 2017 to December 2018.

Inclusion criteria were: 1) at least 18 years old; 2) MRF (4 or more fractured ribs) with bicortical displacement; 4) intractable pain with visual analogue scale >6 after conservative treatment (Nirula et al. 2009).

Exclusion criteria were: 1) serious head trauma with a Glasgow coma scale <14; 2) multiple trauma at body regions outside the chest with an abbreviated injury scale score ≥3; 3) massive hemothorax or injury to the trachea or bronchus that required emergent surgery; 4) mechanically ventilated; 5) dementia; 6) coagulopathy.

### SSRF technique

Our standard SSRF procedure combined video-assisted thoracoscopic surgery (VATS) and open reduction internal fixation (ORIF), using a muscle-sparing approach without thoracotomy (Ali-Osman et al. 2018). Moreover, to prevent iatrogenic injury caused by drilling and/or manipulation, a safe pleural space was generated. For patients with chest tubes, VATS was performed with a 30° 5 mm thoracoscope through the wound of thoracostomy. For patients without chest tubes, a 1 cm port was created along the anterior axillary line at the fifth intercostal space. ORIF was subsequently performed with a non-precontoured universal 3.5 mm metal locking plate (Althausen et al. 2011). Following ORIF, VATS was then utilised to examine screw penetration through the parietal pleura. In the case of air leakage through lung laceration, a second VATS port was created for repair and resection using a linear cutter stapler (Chou YP et al. 2014). Direct VATS visualisation was then used to direct the placement of a chest tube. For patients with unstable sternal fracture, such as segmental fracture or distraction, ORIF of the sternal fracture was performed at the same time using a pre-contoured locking plate (Chou SS et al. 2011). After SSRF, the MRF patients were admitted into the Intensive Care Unit (ICU) with endotracheal tubes in place.

### EPI analgesia

In general, patients who required EPI had a pharmacologic thromboprophylaxis (PTP) hold for 12 h before the procedure as a safe window, followed by PTP provided as 40 mg enoxaparin sodium once per day. EPI was administered as continuous infusion using a fixed electronic infusion device with portless tubing, with patients in a 25° head of bed elevation. EPI analgesia was achieved as a combination of 0.1% bupivacaine with 5 μg/mL fentanyl at 4 mL/h rate for approximately 3 h per day during the course of the study. IS was encouraged ten times per h while patients were awake.

### Group assignment and intervention

The 97 patients were divided using a permutated randomisation method stratified according to their baseline pain scores, with 49 in the EGCG group and 48 in the placebo group. In the EGCG group, patients were administered one capsule containing 100 mg EGCG (oral grade, purity >94%; purchased from Taiyo International, Yamadacho, Japan), twice daily. In the placebo group, patients were administered one capsule containing 100 mg glucose (instead of EGCG), twice daily. Both types of capsules were identical in appearance to mask their contents to both the investigators and patients. Both interventions last for 10 days.

### Endpoints

Primary endpoint was pain score measured using a standard 0-10 numeric rating scale (NRS), with 0 indicating no pain at all and 10 indicating the worst possible pain. Secondary endpoints included IS volume, respiratory rate, and SpO_2_. All assessments were performed at the hospital admission (day 0, baseline) and at the end of intervention (day 10) by bedside nurses who were blind to the group assignment.

### Statistical analysis

Statistical analysis was performed using the Graphpad software version 7. Data in the current study were found to be non-normally distributed therefore shown as median and interquartile range. P-value was calculated as two-sided and *p* < 0.05 was considered to indicate statistically significant difference between groups.

## Results

**Figure 1.**
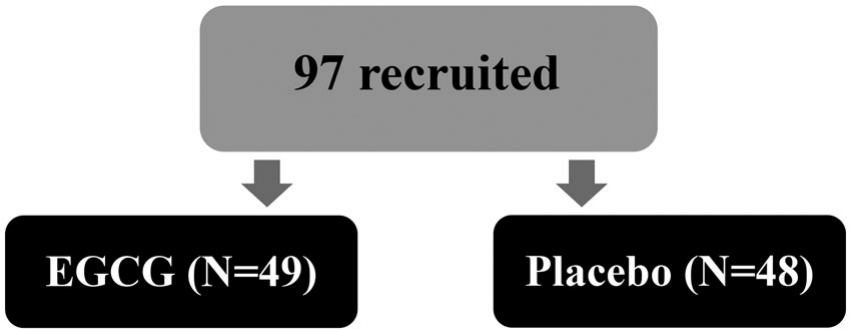
Flow chart of the study.

From January 2017 to December 2018, 97 patients with MRF who underwent SSRF and received EPI analgesia met the inclusion and exclusion criteria. As shown in Figure 1, they were divided into two study groups, with 49 patients in the EGCG group and 48 in the placebo group. Demographic data of patients in the two study groups were listed in Table 1, and no obvious differences were found between the two groups.

**Table 1.** Patient demographics at baseline.

Demographics	EGCG (*N* = 49)	Placebo (*N* = 48)
Age, year, median (range)	41 (24-62)	45 (19-67)
Male, *n* (%)	29 (59%)	32 (69%)
Number of fractured ribs, median (range)	6 (4-9)	7 (5-9)
Length of hospital stay, day, median (range)	13 (10-18)	14 (11-17)

Pain score was the primary endpoint of the current study, which was assessed in the form of NRS. As shown in Table 2, pain scores between day 0 (baseline) and day 10 (end of intervention) were markedly reduced in both groups (*p < 0.05), suggesting the general effectiveness of EPI analgesia in alleviating pain. Importantly, at the end of intervention, pain score of the EGCG group was significantly lower than that of the placebo group (Table 2, #*p* < 0.05), demonstrating the efficacy of EGCG in terms of additional pain relief on top of EPI analgesia.

**Table 2.** Pain scores of patients at baseline (day 0) and end of intervention (day 10).

	EGCG (*N* = 49)		Placebo (*N* = 48)	
	Day 0	Day 10	Day 0	Day 10
Pain scores, median (range)	8 (6-10)	4 (3-6)*	8 (5-10)	6 (4-8)*#

*p < 0.05 intra-group comparison between day 0 and day 10; #*p* < 0.05 inter-group comparison.

As the secondary endpoints, IS volume, respiratory rate and SpO_2_ were also measured (Table 3). IS volume exhibited a similar trend of change as pain score, with apparent improvements seen in both groups from baseline to the end of intervention (Table 3, *p < 0.05), and further improved IS volume was observed at the end of intervention in the EGCG group compared to that in the placebo group (Table 3, #*p* < 0.05). The same effect was also observed in terms of respiratory rate (Table 4), but not in SpO_2_ (Table 5). Although SpO_2_ in both groups were greatly improved from baseline to the end of intervention (Table 5, *p < 0.05), no significantly different change was found in SpO_2_ at the end of intervention between the two study groups (Table 5, $*p* > 0.05).

**Table 3.** Incentive spirometry (IS) volume of patients at baseline (day 0) and end of intervention (day 10).

	EGCG (N = 49)		Placebo (N = 48)	
	Day 0	Day 10	Day 0	Day 10
IS volume, median (range)	713 (365-1254)	1072 (436-1533)*	728 (341-1186)	953 (403-1429)*#

*p < 0.05 intra-group comparison between day 0 and day 10; # *p* < 0.05 inter-group comparison.

**Table 4.** Respiratory rate of patients at baseline (day 0) and end of intervention (day 10).

	EGCG (*N* = 49)		Placebo (*N* = 48)	
	Day 0	Day 10	Day 0	Day 10
Respiratory rate, median (range)	24 (11-38)	15 (12-27)*	25 (10-39)	19 (13-31)*#

*p < 0.05 intra-group comparison between day 0 and day 10; #*p* < 0.05 inter-group comparison.

**Table 5.** Oxygen saturation (SpO2) of patients at baseline (day 0) and end of intervention (day 10).

	EGCG (*N* = 49)		Placebo (*N* = 48)	
	Day 0	Day 10	Day 0	Day 10
SpO2, median (range)	0.94 (0.89-1.00)	0.98 (0.96-1.00)*	0.93 (0.88-1.00)	0.98 (0.95-1.00)*$

*p < 0.05 intra-group comparison between day 0 and day 10; $*p* > 0.05 inter-group comparison.

## Discussion

Multiple previous studies have implicated EGCG in various animal models of pain management. In murine models of chronic neuropathic pain, EGCG was shown to significantly improve pain behaviours, likely mediated by inhibiting Toll-like receptor 4 (Kuang et al. 2012) and blocking expression of nitric oxide synthase (Choi et al. 2012). Moreover, EGCG could also inhibit radiculopathic pain in rats (Krupkova et al. 2014). In a mouse model of bone cancer pain, EGCG exhibited anti-nociceptive functions in the pain progression following metastasis of bone tumour (Li & Zhang 2015). In clinical settings, an ointment containing EGCG as the active component was able to promote wound healing and relieve episiotomy pain in primiparous women (Shahrahmani et al. 2018). In addition, in patients with shoulder pain, EGCG treatment was reported with a potential to relieve rotator cuff tendinopathy-related pain and symptoms (Feng et al. 2019).

Our current study is the first instance to provide experimental evidence, in a clinical setting, that supports an adjuvant beneficial role of EGCG to enhance the effect of EPI analgesia in the pain management after SSRF for MRF patients. In general, EPI analgesia greatly relieved pain scores, which was the primary endpoint, in MRF patients after their SSRF operation. Additional oral administration of EGCG in the 10-day intervention course further reduced pain scores compared to the placebo treatment. As the secondary endpoints, IS volume and respiratory rate were also further improved by EGCG, when combined with EPI analgesia. Although such EGCG-related improvements were absent in terms of SpO_2_, the outcomes in IS volume and respiratory rate implied that EGCG could also improve recovery of MRF patients in functional aspects after SSRF. Sufficient analgesia is reported to improve respiratory mechanics, enhance functional capacity and prevent pulmonary complications (Brown & Walters 2012; Brasel et al. 2017; Fusco et al. 2017). In line with this notion, our results are more clinically relevant, as the primary outcome of pain score was correlated with improved pulmonary functions, thereby demonstrating a promising potential for EGCG to exert additional clinical benefits on top of conventional EPI pain management.

Rib fractures often result from blunt chest trauma and are the main cause of hospital admission. Rib fractures result in pain and disability, and many patients also develop pneumonia and/or chest wall deformity. Previous randomised controlled trials have shown that, SSRF is superior to conservative treatments for patients with flail chest and respiratory failure (Granetzny et al. 2005). Review articles and meta-analyses also showed that SSRF could reduce ICU and hospital length of stay, duration of ventilation, and tracheostomy rate. The indications for SSRF have been well established based on expert consensus.

However, poor pain control means that patients’ active mobilisation is impeded by persistent pain, despite optimised medical treatment with morphine mimetics and non-steroid anti-inflammatory drugs. Locoregional pain relief with para-vertebral blockade or EPI analgesia is ambiguous in the sense that, it cannot be used over a prolonged period, but rib fractures are still unstable and painful at 4-6 days after the initial trauma (Olland et al. 2019). Our 10-day EGCG oral administration, although still considered a short-term intervention, may serve as a less invasive but prolonged adjuvant analgesia option beyond the limitation of EPI analgesia.

Nevertheless, there are several limitations in our current investigation. 1) It was a single centred study; therefore, our prospective results should be verified in a multi-centred study for generality considerations. 2) The sample size was relatively small; therefore, a larger sample pool would definitely bring more sophisticated statistical analysis and facilitate the identification of associated risk factors. 3) No serum factors known to be regulated by EGCG were assessed in the current study; future investigations are needed to identify the potential molecular mechanism underlying the observed benefits of EGCG in this setting.

## Conclusions

In this double-blind, placebo-controlled trial, we have demonstrated that oral EGCG administration could enhance pain management of EPI analgesia for MRF patients after SSRF operation. Our discovery warrants further investigations in order to compare the observed pain-relieving effect of EGCG with other analgesia techniques.

## References

[CIT0001] Ali-Osman F, Mangram A, Sucher J, Shirah G, Johnson V, Moeser P, Sinchuk NK, Dzandu JK. 2018. Geriatric (G60) trauma patients with severe rib fractures: is muscle sparing minimally invasive thoracotomy rib fixation safe and does it improve post-operative pulmonary function? Am J Surg. 216(1):46–51. 29525055 10.1016/j.amjsurg.2018.02.022

[CIT0002] Althausen PL, Shannon S, Watts C, Thomas K, Bain MA, Coll D, O'Mara TJ, Bray TJ. 2011. Early surgical stabilization of flail chest with locked plate fixation. J Orthop Trauma. 25(11):641–647. 22008858 10.1097/BOT.0b013e318234d479

[CIT0003] Brasel KJ, Moore EE, Albrecht RA, deMoya M, Schreiber M, Karmy-Jones R, Rowell S, Namias N, Cohen M, Shatz DV, et al. 2017. Western trauma association critical decisions in trauma: management of rib fractures. J Trauma Acute Care Surg. 82(1):200–203. 27779590 10.1097/TA.0000000000001301

[CIT0004] Brown SD, Walters MR. 2012. Patients with rib fractures: use of incentive spirometry volumes to guide care. J Trauma Nurs. 19(2):89–91. quiz 92-83. 22673074 10.1097/JTN.0b013e31825629ee

[CIT0005] Choi JI, Kim WM, Lee HG, Kim YO, Yoon MH. 2012. Role of neuronal nitric oxide synthase in the antiallodynic effects of intrathecal EGCG in a neuropathic pain rat model. Neurosci Lett. 510(1):53–57. 22249118 10.1016/j.neulet.2011.12.070

[CIT0006] Chou SS, Sena MJ, Wong MS. 2011. Use of SternaLock plating system in acute treatment of unstable traumatic sternal fractures. Ann Thorac Surg. 91(2):597–599. 21256325 10.1016/j.athoracsur.2010.07.083

[CIT0007] Chou YP, Kuo LC, Soo KM, Tarng YW, Chiang HI, Huang FD, Lin HL. 2014. The role of repairing lung lacerations during video-assisted thoracoscopic surgery evacuations for retained haemothorax caused by blunt chest trauma. Eur J Cardiothorac Surg. 46(1):107–111. 24242850 10.1093/ejcts/ezt523 PMC4057012

[CIT0008] Feng H, He Z, Twomey K, Ilaltdinov AW, Leong D, Wang Y, Li J, Gonzalez D, Sun HB. 2019. Epigallocatechin-3-gallate suppresses pain-related and proinflammatory mediators in the subacromial bursa in rotator cuff tendinopathy. Discov Med. 27(147):63–77. 30825883

[CIT0009] Fraser SF, Tan C, Kuppusamy MK, Gukop P, Hunt IJ. 2017. The role of a video-assisted thoracic approach for rib fixation. Eur J Trauma Emerg Surg. 43(2):185–190. 26850079 10.1007/s00068-016-0641-1

[CIT0010] Fusco P, Scimia P, Di Carlo S, Testa A, Luciani A, Petrucci E, Marinangeli F. 2017. Ultrasound-guided serratus plane block and fast-track tracheal extubation in the operating room for thoracic trauma patients: a case report. A A Case Rep. 9(11):305–307. 28767478 10.1213/XAA.0000000000000600

[CIT0011] Galvagno SM, Jr., Smith CE, Varon AJ, Hasenboehler EA, Sultan S, Shaefer G, To KB, Fox AD, Alley DE, Ditillo M, et al. 2016. Pain management for blunt thoracic trauma: a joint practice management guideline from the Eastern Association for the Surgery of Trauma and Trauma Anesthesiology Society. J Trauma Acute Care Surg. 81(5):936–951. 27533913 10.1097/TA.0000000000001209

[CIT0012] Granetzny A, Abd El-Aal M, Emam E, Shalaby A, Boseila A. 2005. Surgical versus conservative treatment of flail chest. Evaluation of the pulmonary status. Interact Cardiovasc Thorac Surg. 4(6):583–587. 17670487 10.1510/icvts.2005.111807

[CIT0013] Haenel JB, Moore FA, Moore EE, Sauaia A, Read RA, Burch JM. 1995. Extrapleural bupivacaine for amelioration of multiple rib fracture pain. J Trauma. 38(1):22–27. 7745650 10.1097/00005373-199501000-00007

[CIT0014] Ho AM, Karmakar MK, Critchley LA. 2011. Acute pain management of patients with multiple fractured ribs: a focus on regional techniques. Curr Opin Crit Care. 17(4):323–327. 21716105 10.1097/MCC.0b013e328348bf6f

[CIT0015] Karmakar MK, Ho AM. 2003. Acute pain management of patients with multiple fractured ribs. J Trauma. 54(3):615–625. 12634549 10.1097/01.TA.0000053197.40145.62

[CIT0016] Krupkova O, Sekiguchi M, Klasen J, Hausmann O, Konno S, Ferguson SJ, Wuertz-Kozak K. 2014. Epigallocatechin 3-gallate suppresses interleukin-1β-induced inflammatory responses in intervertebral disc cells in vitro and reduces radiculopathic pain in rats . Eur Cell Mater. 28:372–386. 25422948 10.22203/ecm.v028a26

[CIT0017] Kuang X, Huang Y, Gu HF, Zu XY, Zou WY, Song ZB, Guo QL. 2012. Effects of intrathecal epigallocatechin gallate, an inhibitor of Toll-like receptor 4, on chronic neuropathic pain in rats. Eur J Pharmacol. 676(1–3):51–56. 22173123 10.1016/j.ejphar.2011.11.037

[CIT0018] Lafferty PM, Anavian J, Will RE, Cole PA. 2011. Operative treatment of chest wall injuries: indications, technique, and outcomes. J Bone Joint Surg Am. 93(1):97–110. 21209274 10.2106/JBJS.I.00696

[CIT0019] Li Q, Zhang X. 2015. Epigallocatechin-3-gallate attenuates bone cancer pain involving decreasing spinal tumor necrosis Factor-α expression in a mouse model . Int Immunopharmacol. 29(2):818–823. 26363974 10.1016/j.intimp.2015.08.037

[CIT0020] Liao S, Kao YH, Hiipakka RA. 2001. Green tea: biochemical and biological basis for health benefits. Vitam Horm. 62:1–94. 11345896 10.1016/s0083-6729(01)62001-6

[CIT0021] Lin SY, Kang L, Chen JC, Wang CZ, Huang HH, Lee MJ, Cheng TL, Chang CF, Lin YS, Chen CH. 2019. (-)-Epigallocatechin-3-gallate (EGCG) enhances healing of femoral bone defect. Phytomedicine. 55:165–171. 30668426 10.1016/j.phymed.2018.07.012

[CIT0022] Lynch N, Salottolo K, Foster K, Orlando A, Koola C, Portillo V, Tanner Ii A, Mains CW, Bar-Or D. 2019. Comparative effectiveness analysis of two regional analgesia techniques for the pain management of isolated multiple rib fractures. JPR. 12:1701–1708. 10.2147/JPR.S198350 PMC6538881 31213882

[CIT0023] Madhurakkat Perikamana SK, Lee SM, Lee J, Ahmad T, Lee MS, Yang HS, Shin H. 2019. Oxidative epigallocatechin gallate coating on polymeric substrates for bone tissue regeneration. Macromol Biosci. 19(4):e1800392. 30645050 10.1002/mabi.201800392

[CIT0024] Nirula R, Diaz JJ, Jr., Trunkey DD, Mayberry JC. 2009. Rib fracture repair: indications, technical issues, and future directions. World J Surg. 33(1):14–22. 18949513 10.1007/s00268-008-9770-y

[CIT0025] Olland A, Puyraveau M, Guinard S, Seitlinger J, Kadoche D, Perrier S, Renaud S, Falcoz PE, Massard G. 2019. Surgical stabilization for multiple rib fractures: whom the benefit?—a prospective observational study. J Thorac Dis. 11(S2):S130–S140. 30906577 10.21037/jtd.2018.10.122 PMC6389565

[CIT0026] Pieracci FM, Majercik S, Ali-Osman F, Ang D, Doben A, Edwards JG, French B, Gasparri M, Marasco S, Minshall C, et al. 2017. Consensus statement: surgical stabilization of rib fractures rib fracture colloquium clinical practice guidelines. Injury. 48(2):307–321. 27912931 10.1016/j.injury.2016.11.026

[CIT0027] Shahrahmani H, Kariman N, Jannesari S, Rafieian-Kopaei M, Mirzaei M, Ghalandari S, Shahrahmani N, Mardani G. 2018. The effect of green tea ointment on episiotomy pain and wound healing in primiparous women: a randomized, double-blind, placebo-controlled clinical trial. Phytother Res. 32(3):522–530. 29235159 10.1002/ptr.5999

[CIT0028] Shen CL, Yeh JK, Cao JJ, Wang JS. 2009. Green tea and bone metabolism. Nutr Res. 29(7):437–456. 19700031 10.1016/j.nutres.2009.06.008 PMC2754215

[CIT0029] Simon BJ, Cushman J, Barraco R, Lane V, Luchette FA, Miglietta M, Roccaforte DJ, Spector R, Group E, EAST Practice Management Guidelines Work Group. 2005. Pain management guidelines for blunt thoracic trauma. J Trauma. 59(5):1256–1267. 16385313 10.1097/01.ta.0000178063.77946.f5

[CIT0030] Solberg BD, Moon CN, Nissim AA, Wilson MT, Margulies DR. 2009. Treatment of chest wall implosion injuries without thoracotomy: technique and clinical outcomes. J Trauma. 67(1):8–13. 19590301 10.1097/TA.0b013e3181a8b3be

